# An Extraordinary Case at Bradford

**Published:** 1915-11-13

**Authors:** 


*To the Editor of* THE HOSPITAL.

### An Extraordinary Case at Bradford.

Although unsigned, we publish the following letter, believing it to be *bona fide*:

*To the Editor of* THE HOSPITAL.

SIR,—In reference to your remarks in last week's issue of THE HOSPITAL *re* child dying from effect of bottle burns.

In the interests of this staff we ask you to make it more plain that the regrettable accident did not happen at the Fever Hospital—which has always been known as the City Hospital—but at the Eye and Throat Clinic. This latter is certainly situated in the City Fever Hospital grounds; it was opened temporarily we believe, but it has its separate matron, nursing staff, and administration.

We feel that the value of our experience here, also of the certificate of training, would be seriously affected if, through your article and a confusion in names, we became known throughout the hospital world as "a house that badly needs setting in order."—Yours, etc.,
TWENTY-EIGHT MEMBERS OF THE NURSING STAFF.
City Hospital, Bradford.
November 9, 1915.

